# Aligning Event-Related Potentials with Transcranial Alternating Current Stimulation for Modulation—a Review

**DOI:** 10.1007/s10548-024-01055-1

**Published:** 2024-04-30

**Authors:** Cindy Boetzel, Heiko I. Stecher, Christoph S. Herrmann

**Affiliations:** 1 Experimental Psychology Lab, Department of Psychology, European Medical School, Cluster for Excellence “Hearing for All”, Carl Von Ossietzky University, Ammerländer Heerstr. 114 – 118, 26129 Oldenburg, Germany; 2 Neuroimaging Unit, European Medical School, Carl Von Ossietzky University, Oldenburg, Germany; 3 Research Center Neurosensory Science, Carl Von Ossietzky University, Oldenburg, Germany

**Keywords:** Transcranial alternating current stimulation, ERP-aligned tACS, Event-related potential, Non-invasive brain stimulation, Event-related oscillations

## Abstract

This review aims to demonstrate the connections between event-related potentials (ERPs), event-related oscillations (EROs), and non-invasive brain stimulation (NIBS), with a specific focus on transcranial alternating current stimulation (tACS). We begin with a short examination and discussion of the relation between ERPs and EROs. Then, we investigate the diverse fields of NIBS, highlighting tACS as a potent tool for modulating neural oscillations and influencing cognitive performance. Emphasizing the impact of tACS on individual ERP components, this article offers insights into the potential of conventional tACS for targeted stimulation of single ERP components. Furthermore, we review recent articles that explore a novel approach of tACS: ERP-aligned tACS. This innovative technique exploits the temporal precision of ERP components, aligning tACS with specific neural events to optimize stimulation effects and target the desired neural response. In conclusion, this review combines current knowledge to explore how ERPs, EROs, and NIBS interact, particularly highlighting the modulatory possibilities offered by tACS. The incorporation of ERP-aligned tACS introduces new opportunities for future research, advancing our understanding of the complex connection between neural oscillations and cognitive processes.

## Introduction

The human brain is a fascinating biological system, which orchestrates the most fundamental bodily functions, such as respiration and heartbeat, up to the underpinnings of advanced mathematical and physical endeavors. The brain’s activity manifests in various ways, making a distinction between continuous brain activity and transient brain activity. Continuous brain activity encompasses neural processes that persist over time, involving processes like maintaining attention, performing complex cognitive tasks or maintaining a certain mental state for a longer period of time. In contrast, transient brain activity occurs in response to distinct events or specific stimuli. An event-related potential (ERP) is an important example of such stimulus-driven transient brain activity. However, one should not stringently separate continuous and transient brain activity, as emerging evidence suggests a profound interaction between these two features of neural dynamics. In this review article, we aim to outline the relationship between ERPs and event-related oscillations (EROs). Moreover, we will shed light on the innovative domain of transcranial electric stimulation (tES) methods that have demonstrated the ability to modulate ERPs. In particular, we will explore the non-invasive transcranial alternating current stimulation (tACS) method, with a focus on its potential to influence ERPs. Additionally, we will present new approaches for time-critical stimulation of distinct ERP components (ERP-aligned tACS).

## Event-Related Potentials (ERPs)

The ERP comprises positive and negative voltage deflections such as P1, N1, P2, N2, and P3, classified by polarity and timing and can reliably be evoked by sensory stimuli of different modalities such as visual (Eischen and Polich 1994; Ravden and Polich 1998), auditory (Falkenstein et al. 1993) or tactile (Eimer et al. 2002) stimuli. The early components reflect basic processing and are mainly modulated by stimulus properties, while later components most likely reflect higher cognitive processes and are modulated by top down mechanisms such as attention and expectation. While there are many more specific components such as N170, N400, P600, Mismatch Negativity, Error Potentials etc. these have not been a subject of investigation in the context of tACS-research and are therefore not considered in this review. The distinct deflections of an ERP are characterized by their morphology, i.e. shape, topography, i.e. scalp distribution, and time of occurrence. They serve as indicators of information processing within the brain (Luck 2014). The generation of ERPs remains a topic of debate, with two models proposed. The first model, the additive power model, suggests that stimuli elicit responses from neural populations with fixed polarity and latency after each presentation of a stimulus or event. These responses are independent from and additive to ongoing brain activity and averaging many of these event-related responses produces the ERP (Sauseng et al. 2007). The second model, the phase-reset model, suggests that ongoing brain activity undergoes a phase reset in response to every stimulus or event, and averaging these phase-coherent rhythms produces the ERP (Jansen et al. 2003; Sauseng et al. 2007). These models are not mutually exclusive, and both are thought to contribute to the generation of the ERP, at least partially (Min et al. 2007). This assumption is supported by the study of Min et al. (2007), who showed dissociable EEG dynamics of total and evoked alpha activity, indicating that not only a phase reset in the alpha band generates the ERP, but that there is also an additive event-related neuronal response involved. Event-related brain activity can be analyzed in the time, frequency and time–frequency domains. Displaying the measured brain activity in the time-domain creates the conventional averaged ERP waveform. Transforming the signal of the ERP to the frequency domain, reveals peaks around 2–3 Hz but also activity in a higher frequency range (~ 10 Hz). For the sake of completeness, it should be mentioned that under certain circumstances, such as distinct spatial frequencies of the stimuli, also higher frequencies, e.g. in the gamma frequency range can occur (see, e.g. Fründ et al. 2007). Combining both domains to the time–frequency domain reveals the temporal dynamics of the spectral components over time. These time–frequency analyses show that the power of the oscillatory activity increases in response to the stimulus or event (Herrmann et al. 2014). This close relationship between brain oscillations and ERPs leads to the assumption that ERPs are a superposition of synchronized oscillatory brain activity (Brandt 1997; Klimesch et al. 2004; Makeig et al. 2002), which is called event-related oscillation (ERO; phase-coherent rhythms).

## ERPs / EROs and Transcranial Current Stimulation

Two distinct categories of EROs are observed in response to sensory or cognitive stimulation: evoked and induced EROs. Induced EROs may exhibit phase shifts from one trial to another, indicating that while some trials reveal peaks and troughs at identical latencies, there are cases in which a peak in one trial may temporally overlap with a trough in another trial or vice versa. Consequently, when averaging the signal across multiple trials, this phase variability can lead to the cancellation of positive and negative deflections within the averaged ERP. On the other hand, when most trials consistently exhibit peaks and troughs of the respective EROs at the same latency, and these oscillations have a strong phase-locking to the onset of the stimulus, they are classified as evoked EROs (Herrmann et al. 2014). Previous Studies have shown that oscillatory activity with different frequencies are involved in the generation of the ERP. Thus, the early components, such as the P1 and N1 are driven by oscillatory activity in the alpha (8—13 Hz) and high theta (4 – 7 Hz) frequency band (Klimesch et al. 2004, 2007), while the later component, the P3, is rather driven by oscillatory activity in the delta/theta frequency band (Demiralp et al. 1999). Thus, the P3 component could be thought of as a positive half-wave in the delta/theta frequency domain, with the phase of oscillatory activity related to the stimulus onset. ERPs and EROs are of great interest in scientific research because they are well suitable for the analysis of temporal-spatial dynamics of several cognitive processes and interestingly, multiple neurodevelopmental and psychiatric disorders such as ADHD (Kaiser et al. 2020; Papp et al. 2020; Wiersema et al. 2009), schizophrenia (Adams et al. 1993; Guillaume and Thomas 2021) or Alzheimer’s disease (Park et al. 2001; Tautvydaitė et al. 2022) show alterations in different features. For example, Kaiser et al. (2020) showed in a meta-analysis that ADHD patients reveal alterations in different ERP components and their features (latency and amplitude) as compared to healthy controls. Since these alterations occur relatively systematically in various diseases (for a meta-analysis of the N170 in schizophrenia patients, see McCleery et al. 2015 or for a meta-analysis of the P300 in ADHD patients, see Szuromi et al. 2011), and they are often associated with the expression of symptoms, they represent a desirable target for treatment methods. While pharmaceutical treatments are often associated with many side effects (Schachter et al. 2001; Wynchank et al. 2017; for a review regarding e.g. the vascular system, see Kahl et al. 2018), invasive stimulation methods are accompanied by the well-known risk of surgeries. Hence, non-invasive brain stimulation methods such as transcranial electric stimulation (tES), provide an advantageous treatment alternative. TES acts at a sub-threshold level and thus alters the probability of spiking depending on the phase of stimulation (Reed and Cohen Kadosh 2018) and represents an inexpensive alternative with few side effects (Matsumoto and Ugawa 2017).

In the past, various tES methods have been investigated for their capability to modulate ERPs. In the context of transcranial direct current stimulation (tDCS), the existing literature presents divergent outcomes concerning its impact on ERPs. These outcomes exhibit inconsistency among studies conducted by Antal et al. (2004), Chen et al. (2023), Cunillera et al. (2016), Lafontaine et al. (2013), Moezzi et al. (2023) or Zaehle et al. (2011), all of which report a modulatory effect of tDCS on ERPs. Conversely, certain investigations, including those by Kunzelmann et al. (2018) and Splittgerber et al. (2021), have observed an absence of modulatory influence exerted by tDCS on the ERP.

However, based on the assumption that ERPs are generated at least in part by a superposition of EROs, in the following we will focus on the transcranial alternating current stimulation (tACS) method, as tACS is a stimulation method especially suitable for targeting endogenous brain oscillations.

### The Relation Between tACS and ERPs

Transcranial alternating current stimulation (tACS) represents a non-invasive method of brain stimulation involving the application of a sinusoidal waveform with gradual voltage oscillating from positive to negative. Several human studies demonstrated the efficacy of tACS (Helfrich et al. 2014; Zaehle et al. 2010; for a review, see Thut et al. 2011). Although the precise mechanism underlying the action of tACS is still subject of investigation, convincing evidence from animal and computational studies (Johnson et al. 2020; Krause et al. 2022; Vieira et al. 2020; Wischnewski et al. 2024) suggest that tACS entrains intrinsic brain oscillations in a frequency-specific manner by aligning them with the frequency of the externally applied alternating current. In a study conducted by Helfrich et al. (2014) the application of 10 Hz tACS over the parieto-occipital cortex revealed a significant increase in 10 Hz alpha activity within the parieto-occipital cortex. An alternative conceivable mechanism of action involves the impact of tACS on the spike time dependent plasticity (Vossen et al. 2015; for a review, see Vogeti et al. 2022). In their study, Vossen et al. (2015) discovered that the tACS effect they successfully reproduced from prior research was not primarily attributable to entrainment. Instead, their findings imply that plasticity mechanisms within oscillatory circuits were the key factors contributing to the observed results. Although the precise mechanism is subject of ongoing debate, there have been multiple examples in the past where the modulatory effect of tACS on endogenous brain oscillations has been demonstrated (Kasten and Herrmann 2017; Wischnewski et al. 2019; Zaehle et al. 2010). Kasten and Herrmann (2017), for instance, reported an enhancement of alpha power following tACS application in the individual alpha frequency. Likewise, Wischnewski et al. (2019) exhibited that 20 Hz beta tACS applied over the primary motor cortex induced elevated cortical excitability and beta power, persisting for at least 60 min post-stimulation. Once more, the outcomes regarding the influence of tACS on brain oscillations exhibit a certain degree of variability. Nevertheless, there is growing evidence supporting the notion that active manipulation of brain activity through tACS is achievable. It is important, however, to consider various individual factors, including anatomy, mental state and stimulation intensities (Kasten et al. 2019).

Given that tACS is well suited to modulate brain oscillatory activity and that oscillations have a clear contribution in the generation of ERPs, it is reasonable to use tACS to modulate ERPs. The question of the feasibility of modulating distinct components or the whole of ERPs, has gained growing interest within the present research. We compiled a list of all tACS studies that attempted to specifically modulate individual ERP-components using tACS (see Table 1 for an overview—PUBMED search terms: “transcranial AND alternating AND current AND stimulation AND event AND related AND potentials”). We focused on classical ERP components originating from visual tasks, though it should be mentioned that there is also a single study on the effects of tACS in tactile components (Sliva 2018).

**Table 1:** Overview of tACS studies with the goal to modulate ERP-components outlined in the review

Study	Analyzed ERP components	Stimulation Frequency	Stimulation Intensity	Stimulation Duration	Electrode Montage	Task	Stimulation effects on ERP
Non-aligned continuous tACS							
Hu et al. (2021)	P2, P3	10 Hz, sham	1.5 mA	20 min	prefrontal Lobe (F3, F4)	emotional face oddball	increased P2, increased P3 amplitude following stimulation
Jaušovec and Jaušovec (2014)	P1, N1, P3	IAF-5 Hz, sham	250 µA below perceptual threshold, (range = 1000 -2250 μA)	15 min	left parietal/frontal brain (P3, F3)	visual-array comparison	decreased P3 amplitude following stimulation
Li et al. (2023)	P2	6 Hz, 70 Hz, sham	> 0.5 mA, below perceptual threshold	20 min	left inferior frontal gyrus (FC5; F3, F7, C3, C7)	frequency-altered feedback voice	increased P2 amplitude by theta and gamma stimulation
Liu et al. (2022)	P2	IAF, 10 Hz, sham	1.5 mA	20 min	prefrontal Lobe (F3, F4)	emotional face oddball	increased P2 amplitude following stimulation
Nakazono et al. 2020	P1, N1	10 Hz, 20 Hz, sham	1 mA	20 min	parietal Lobe (Pz, Oz)	pattern reversal, focal flash	N75-P100 complex increase following alpha stimulation
Pahor and Jaušovec (2017)	P1, N1	ITF, IGF, sham	250 µA below perceptual threshold, (range = 1250 -2000 µA)	15 min	bilateral frontal (F3, F4), bilateral parietal (P3, P4), left fronto-parietal (F3, P3) and right-fronto parietal (F4, P4)	figural change detection, figural 2-back	change detection task: decreased P3 latencies
N-back task: P1 amplitude increase following theta stimulation							
Wischnewski et al. (2021)	P3a, P3b, FRN	2.5 Hz, 5 Hz, sham	1 mA	12 min	frontal cortex (Fpz-Afz, Cz)	decision-making	decreased P3a and P3b amplitudes following theta stimulation
ERP alligned tACS							
Boetzel et al. (2023)	P3	IDF/ITF matched to P3 frequency, shunt-control	1 mA per electrode pair	max 30 min	centro-parietal region (FC3h, FC5, C3h, C5, CP3h, CP5 vs FC4h, FC6, C4h, C6, CP4h, CP6)	visual oddball-like (Gabor)	increased P3 amplitude following stimulation
Dallmer-Zerbe et al. (2020)	P3	IDF/ITF matched to P3 frequency, sham-control	1 mA divided on 6 electrode pairs	20 min	centro-parietal region (C3, C4, CP3, CP4, P3, P4 vs T7, T8, TP7, TP8, P7, P8)	visual oddball (x-o)	increased P3 amplitude following stimulation
Kannen et al. (2022)	P3	IDF/ITF matched to P3 frequency, shunt-control	1 mA	20 min	centro-parietal region (C1/C2 vs C5/C6)	visual oddball-like (Gabor)	no P3 increase following stimulation; N700 amplitude increase
Popp et al. (2019)	P3	IDF/ITF matched to P3 frequency, sham-control	1 mA divided on 6 electrode pairs	20 min	centro-parietal region (C3, C4, CP3, CP4, P3, P4 vs T7, T8, TP7, TP8, P7, P8)	visual oddball (x-o)	no stimulation effects on ERP components

*IAF* individual alpha frequency, *IDF* individual delta frequency, *ITF* individual theta frequency.

### Early ERP Components and tACS

#### P1 and N1

The visual P1, occurring about 60 – 100 ms after the stimulus onset (Luck 2014), is thought to originate from the extrastriate cortex / fusiform gyrus (Herrmann and Knight 2001) irrespective of the task the participant is performing (Luck 2014), suggesting its stimulus-driven nature. The N1, occurring ~ 80 – 120 ms after the stimulus onset (Joos et al. 2014), is thought to be also be generated in extrastriate occipital cortex. In the auditory domain, the P1 and N1 are usually referred to as the P50 and N100, respectively, and stem mainly from the auditory cortex / superior temporal lobe (Korzyukov et al. 2007; Näätänen and Picton 1987).

While the P1 relates to spatial selective attention and early sensory processing (Rugg et al. 1987), the N1 is linked to exogenous attention allocation (Natale et al. 2006) and the processing of any unexpected stimuli.

In a study conducted by Pahor and Jaušovec (2017), an investigation was undertaken to discriminate the effect of tACS in the theta and gamma frequency range upon distinct components of the ERP. The study had a comprehensive design, incorporating four different stimulation sites and two stimulation frequencies. These distinct frequencies were administered in combination with various behavioral tasks. The findings of the study revealed that the application of tACS neither significantly altered the amplitudes nor the latencies of the ERP components elicited by the change detection task. However, in the n-back task, theta tACS increased the P1 amplitude when bilateral theta tACS was applied over the parietal cortex. Furthermore, the application of bilateral frontal theta tACS resulted in a comparable increase of the P1 amplitudes. Jaušovec and Jaušovec (2014) demonstrated that the application of theta tACS to twenty-four participants exhibited negligible impact on the P1 latency or amplitude in a visual-array comparison task. Given the similarity in stimulation frequency in both studies, the variance in results may be caused by the different stimulation sites. In the study conducted by Pahor and Jaušovec (2017), effects on the P1 amplitude were exclusively evident following bilateral frontal and parietal stimulations, while unilateral frontal or parietal stimulations failed to affect the early ERP components. In the study of Jaušovec and Jaušovec (2014) the target electrode was placed unilaterally over the left parietal cortex (electrode P3) or the left frontal cortex (electrode F3). Hence, a bilateral stimulation might be needed to modulate the activity of the network responsible for the generation of the P1 component. In a separate investigation conducted by Nakazono et al. (2020), participants underwent tACS employing either alpha or beta frequencies, as well as a sham condition, targeted at the parietal cortex (Cz – Oz montage). The authors reported that alpha tACS increased the N75-P100 complex amplitude, while this effect was not observed in the P100-N145 complex when compared to the effects of beta tACS. Remarkably, the findings of this study did not indicate an increase in alpha power subsequent to alpha-stimulation; rather, they revealed an elevated inter-trial coherence of alpha oscillations.

These findings are congruent with the phase-reset model, as an increased inter-trial coherence signifies a consistent phase alignment of the alpha oscillatory activity in each trial and averaging these trials is anticipated to yield increased early ERP components. Nonetheless, the observed effects on P1 amplitude due the theta-frequency stimulation was not congruent with the expectation (Pahor and Jaušovec 2017). It is important to consider that the influence of sub-harmonics and harmonics related to the stimulation frequency on neuronal activity has been established in simulation studies (Herrmann et al. 2016; Hutt et al. 2018; Lefebvre et al. 2017; Negahbani et al. 2018). Therefore, the stimulation effect observed might be attributed to harmonics of the applied theta-frequency stimulation.

#### P2 and N2

Following the P1 and N1 components, the P2 component is evoked, primarily involved in cognitive functions such as working memory and semantic processing, and exhibits posterior cortical origins. The P2 component is frequently linked to the N1 component within the N1-P2 complex but can also appear independently (for a review, see Crowley and Colrain 2004). The P2 is predominantly evoked by auditory stimuli but can also be elicited by visual and somatosensory stimuli, and is also involved in attention-processes, salience detection and reward encoding (Potts 2004). It responds to both attended and unattended stimuli. A subsequent neural component, the N2, arises in response to repetitive non-target stimuli. Its amplitude increases when deviant stimuli appear in a series of non-target trials (Luck 2014). The N2 may potentially reflect inhibitory processes (Eimer 1993), conflict monitoring, or the level of conflict between required and prepared responses (Donkers and van Boxtel 2004; McLoughlin et al. 2010). For instance, N2 exhibits greater amplitudes in no-go trials compared to go-trials, with the most pronounced amplitude observed in central regions, notably at electrode site Cz (Eimer 1993). The P2-N2 complex is driven by delta and theta frequency brain oscillations (Marturano et al. 2020).

A number of studies have explored the impact of tACS on both the P2 and N2 components. Hu et al. (2021) applied 10 Hz tACS or sham to the prefrontal cortex of forty-four healthy participants during a face oddball task and showed significantly increased P2 amplitudes for positive and negative target stimuli. This phenomenon was not evident within the sham group. In a recent study conducted by Li et al. (2023), high-definition (HD)-tACS was administered at 6 Hz or 70 Hz and in respective sham conditions over the left inferior frontal gyrus (IFG). A total of twenty participants underwent stimulation while performing a frequency-altered feedback task. The study's results revealed significantly larger P2 amplitudes when subject to either 6 Hz or 70 Hz HD-tACS over the left IFG in comparison to the sham HD-tACS condition. Another study, conducted by Liu et al. (2022), applied individual alpha frequency (IAF), 10 Hz or sham-tACS bilaterally to the dorsolateral pre-frontal cortex (dlPFC) of 79 participants. Their study revealed a significant increase in P2 amplitude after active-tACS. Moreover, a noteworthy positive association was identified between alpha power and P2 amplitude.

### General tACS Effects on the P3 Component

The ERP’s most prominent component is the P3, which is entirely task-dependent and appearing as a substantial positive deflection approximately 300–650 ms post-stimulus onset. It comprises several subcomponents, namely, the early P3a, novelty P3 and late P3b, which may vary in prominence based on the task requirements (Barry et al. 2020; Courchesne et al. 1975; Squires et al. 1975). For the purpose of the subsequent discussion, the P3b will be referred to as P3. The P3 is closely associated with the allocation of cognitive resources (Polich 2007) and is characterized by three key parameters: amplitude, latency, and topography. Manipulations of the task can exert a significant influence on P3 amplitude. The P3’s latency is linked to processes encompassing stimulus categorization, response execution, and selection processes (Polich 2007). The topography depends on the type of P3, P3a being more frontally distributed while P3b is located more posteriorly.

Numerous studies have explored the influence of transcranial alternating current stimulation (tACS) on the P3 component and its consequential effects on behavioral outcomes. For example, in their study, Jaušovec and Jaušovec (2014) reported that theta tACS did not elicit a statistically significant effect on P3 amplitude; however, it did lead to a significant reduction in P3 latency. Considering that P3 latency serves as an indicator of classification speed, the authors hypothesize that the application of theta tACS may have increased the participants’ ability to allocate the necessary cognitive resources for successful completion of the working memory task, when the stimulation electrode was placed over the parietal cortex (Jaušovec and Jaušovec 2014). Comparable findings were reported by Pahor and Jaušovec (2017), where decreased P3 latencies were observed in comparison to sham condition. They argued that the decreased latencies might signify accelerated item matching. However, in contrast to the earlier findings of Jaušovec and Jaušovec (2014), these authors also demonstrated an increase in P3 amplitude following the application of theta tACS when it was targeted at the right frontoparietal cortex. It was noted that the P3 amplitude at the electrode position Pz exhibited a positive correlation with the task accuracy. Furthermore, Hu et al. (2021) demonstrated, in addition to the observed enhancement in P2 amplitude, an increase in P3 amplitudes subsequent to the application of alpha tACS over the prefrontal cortex. The authors proposed that tACS may effectively regulate attention allocation during emotional processing in healthy individuals, enhance the effect of negative emotion cognition, and influence the cognitive aspects of emotional processing within the brain.

In contrast to the observed elevating effects of theta tACS, Wischnewski and Schutter (2017) reported contrasting findings. In their study, participants underwent a 12-min application of delta (2.5 Hz), theta tACS (5 Hz) and a sham tACS condition, all administered over the frontal cortex. The results indicated that the application of delta tACS decreased oscillatory power within the 1.5 Hz to 2.5 Hz frequency range, in comparison to both the theta tACS and sham conditions. The reduction in oscillatory power was particularly prominent slightly below the stimulation frequency. Conversely, the application of theta tACS did not yield a significant difference in evoked power when compared to the sham condition. Importantly, the decrease in delta-evoked power did not correspond to any notable alterations in behavioral outcomes. A reduction in delta power subsequent to delta tACS could result in decreased P3 amplitudes if the phase-reset model is valid. Nevertheless, since the authors did not provide information on P3 amplitudes, we are unable to comment on the potential modulation of P3 amplitudes induced by the applied stimulation. In a further study of Wischnewski et al. (2021), the authors administered theta tACS over the frontal cortex of 24 participants engaged in a decision-making task, and they observed a significant decrease in the amplitude of the P3b component following theta tACS compared to sham tACS. The authors postulate that this finding may indicate a diminished engagement of participants in context updating, specifically, the process of learning from reward and punishment feedback. Thus, while the P3b component was indeed affected by theta tACS in comparison to sham, the directionality of this effect, namely a reduction rather than an increase, may have prevented the emergence of notable behavioral changes.

These studies employed continuous tACS regardless of the timing of the stimulus presentation with respect to the phase of the sinusoidal tACS signal. This methodology is justified, as increased amplitude of the stimulated frequency band would inherently result in increased amplitude of the individual ERP components according to a phase reset of an oscillation within each trial. Considering the findings of previous research, indicating that both the additive power and the phase reset model are involved in the generation of an ERP (Fell et al. 2004; Min et al. 2007; Sauseng et al. 2007), this non-aligned continuous tACS method would only affect phase-reset dependent portion of the ERP-generation. An alternative approach that would affect both phase-reset and additive-power contributions would seem beneficial.

### Targeted P3 Modulation with ERP-Aligned tACS

**Fig. 1:**
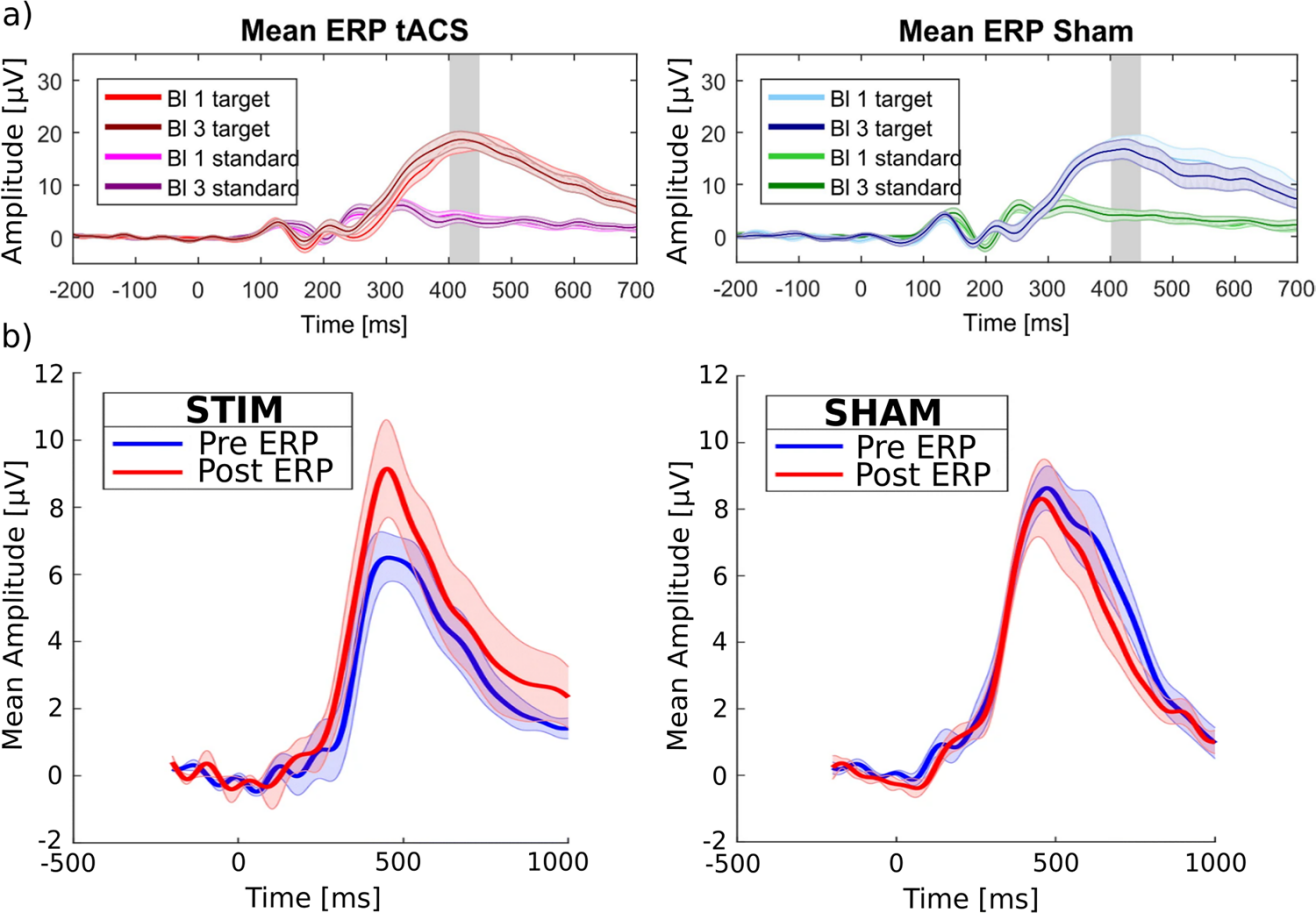
Electrophysiological results of **a**) Popp et al. (2019) in healthy participants and b) Dallmer-Zerbe et al. (2020) in a cohort of ADHD patients. In healthy participants (a), the P3 component at the Pz does not show a significant increase after aligned tACS for either targets or standard stimuli when compared to the sham condition. **b**) In patients with ADHD, there was a significant increase in the P3 amplitude after aligned tACS, whereas no such increase was observed after sham stimulation. Adapted figure from a) Popp et al. (2019) and b) Dallmer-Zerbe et al. (2020)

**Fig. 2:**
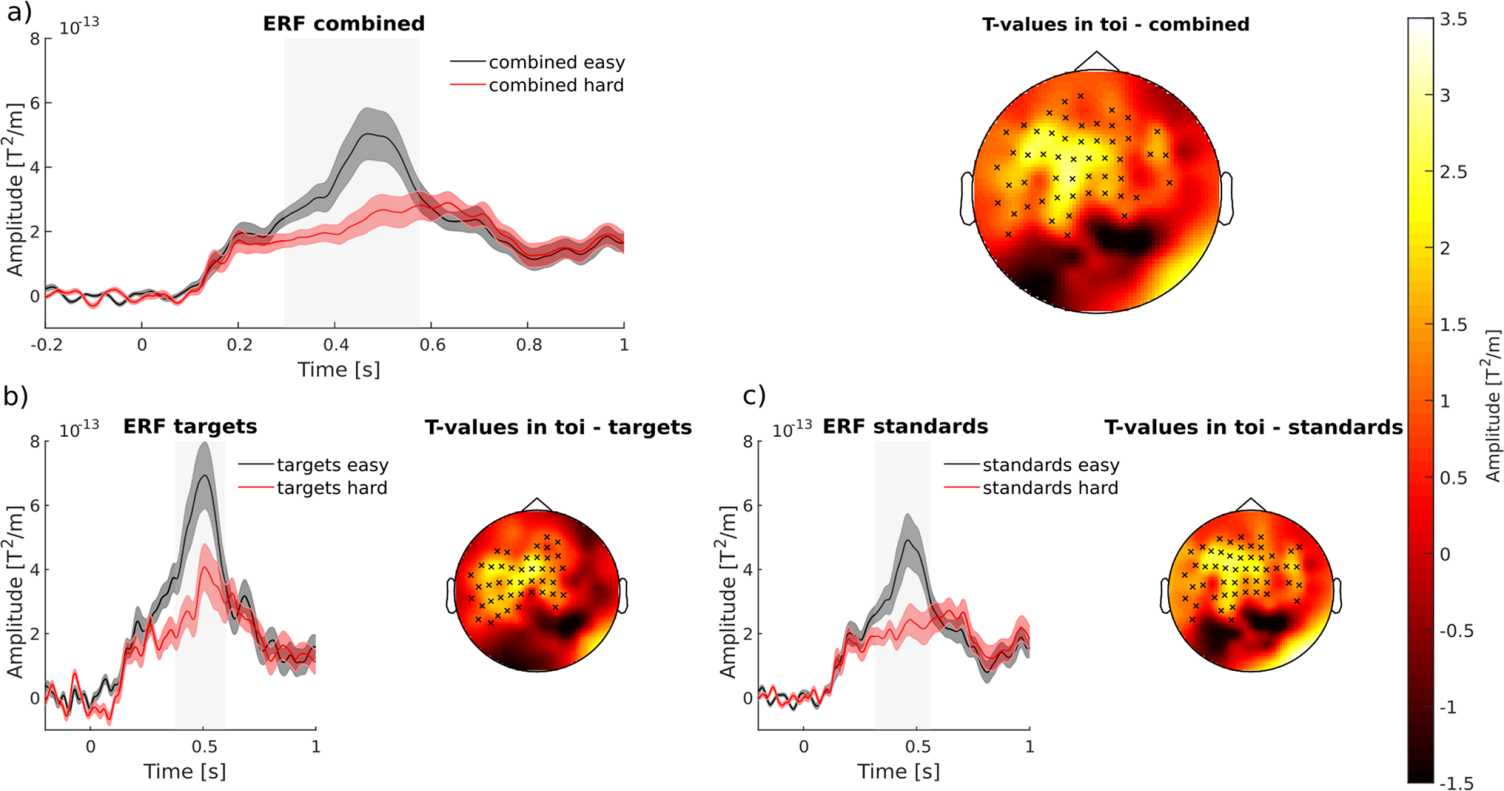
Visualization of cluster-based permutation test (CBPT) of difficulty effect. **a**) Combined ERFs of gradiometers showing a significant decrease in the P3m in the hard condition (red line) as compared to the easy condition (black line). Channel included in the average ERF are indicated by small crosses in topography top right. Shaded error bars represent the standard error of the mean. Grey bars indicate the TOI (time of interest), which is the time interval in which the CBPT revealed significant differences between the easy and hard condition. The topography (top right) shows the t-values resulted from the CBPT averaged over the TOI. Black crosses indicate the channel, associated with the significant cluster. **b**) ERFs and topography with t-values for target trials. c) ERFs and topography with t-values for standard trials. Figure from Boetzel et al. (2024)

**Fig. 3:**
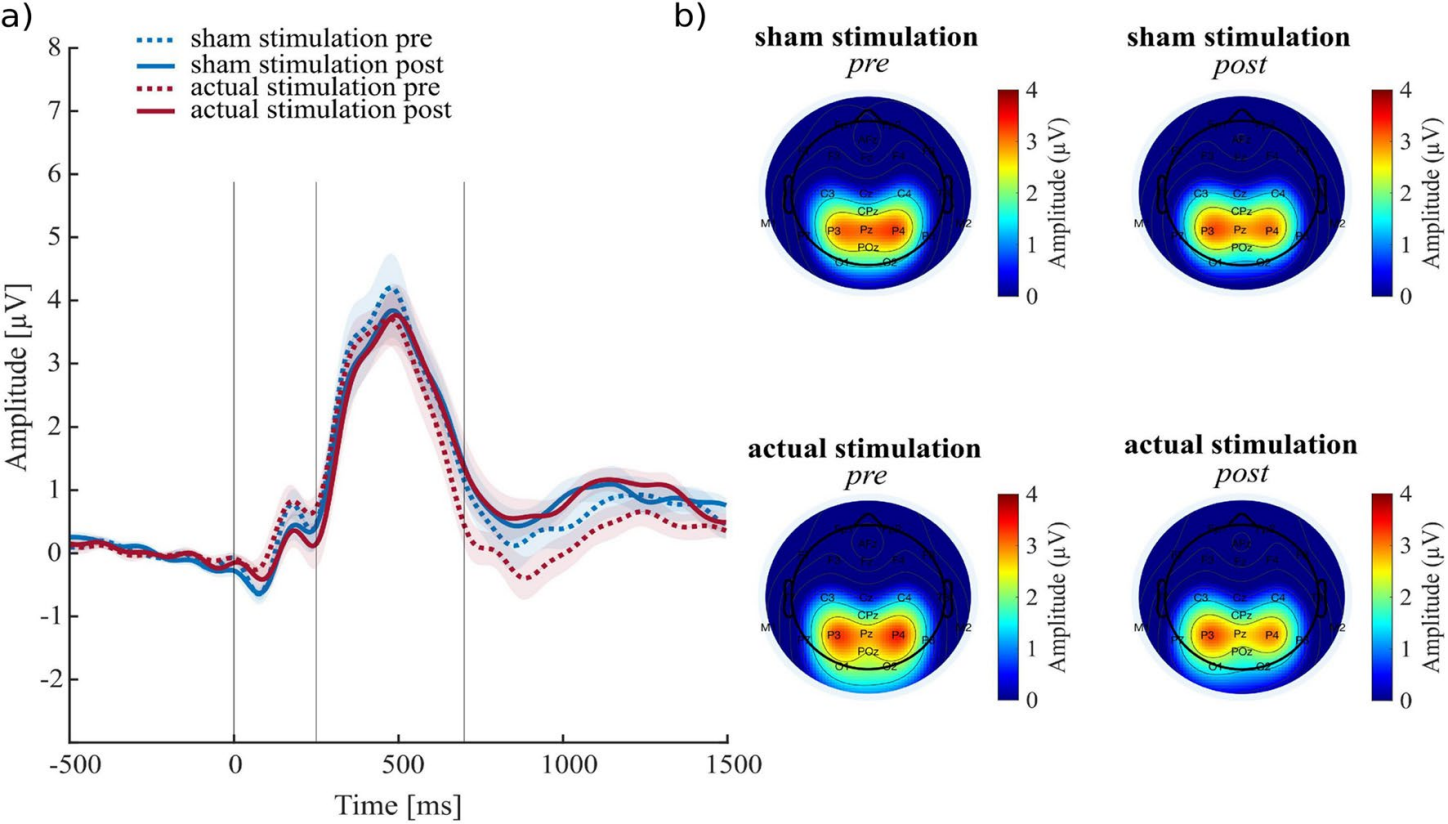
Electrophysiological results of the study conducted by Kannen et al. (2022). **a**) Pre- and post-ERPs for either stimulation or sham condition. No stimulation effect was found for the P3 amplitude but in the time window between ~ 700 to 1000 ms after stimulus onset. **b**) Topographical maps before and after sham and stimulation conditions, including data 250 – 550 ms following stimulus onset, showing no modulation in brain activity post-stimulation compared to the sham condition. Adapted Figure from Kannen et al. (2022)

**Fig. 4:**
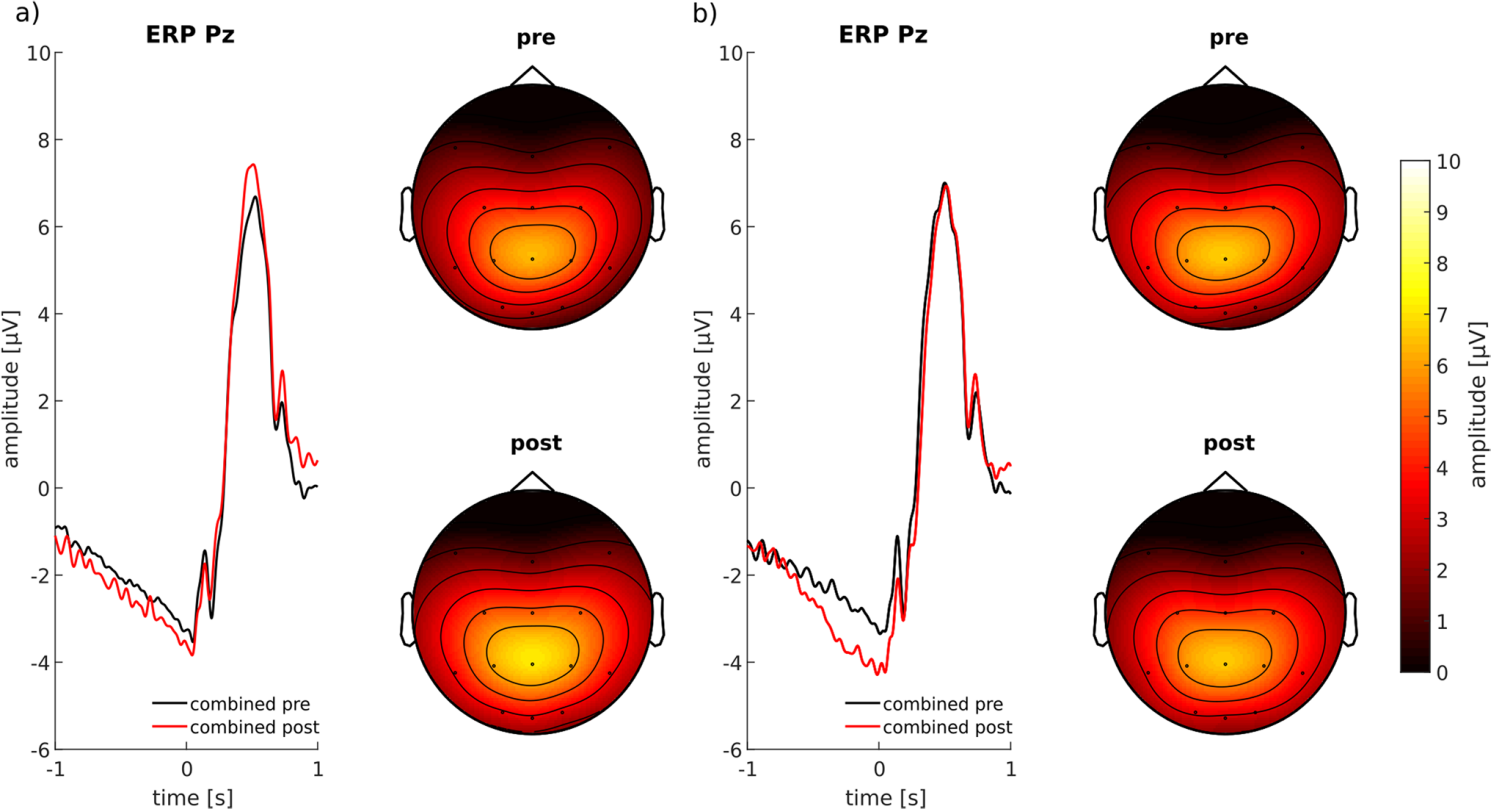
Electrophysiological results of the study conducted by Boetzel et al., (2023). **a**) On the left: Pre- and post-ERPs of the stimulation condition with a significantly increased P3 amplitude after stimulation. a) On the right: Topographies of the brain activity in a time interval ± 50 ms around the peak of the P3 with higher amplitudes in the post-block (red line) as compared to the pre-block (black line). **b**) On the left: ERPs and on the right: topographies of the activity in the pre- and post-block of the control condition, with no significant differences between pre- and post-block. Adapted Fig. 3 of Boetzel et al. (2023)

**Fig. 5:**
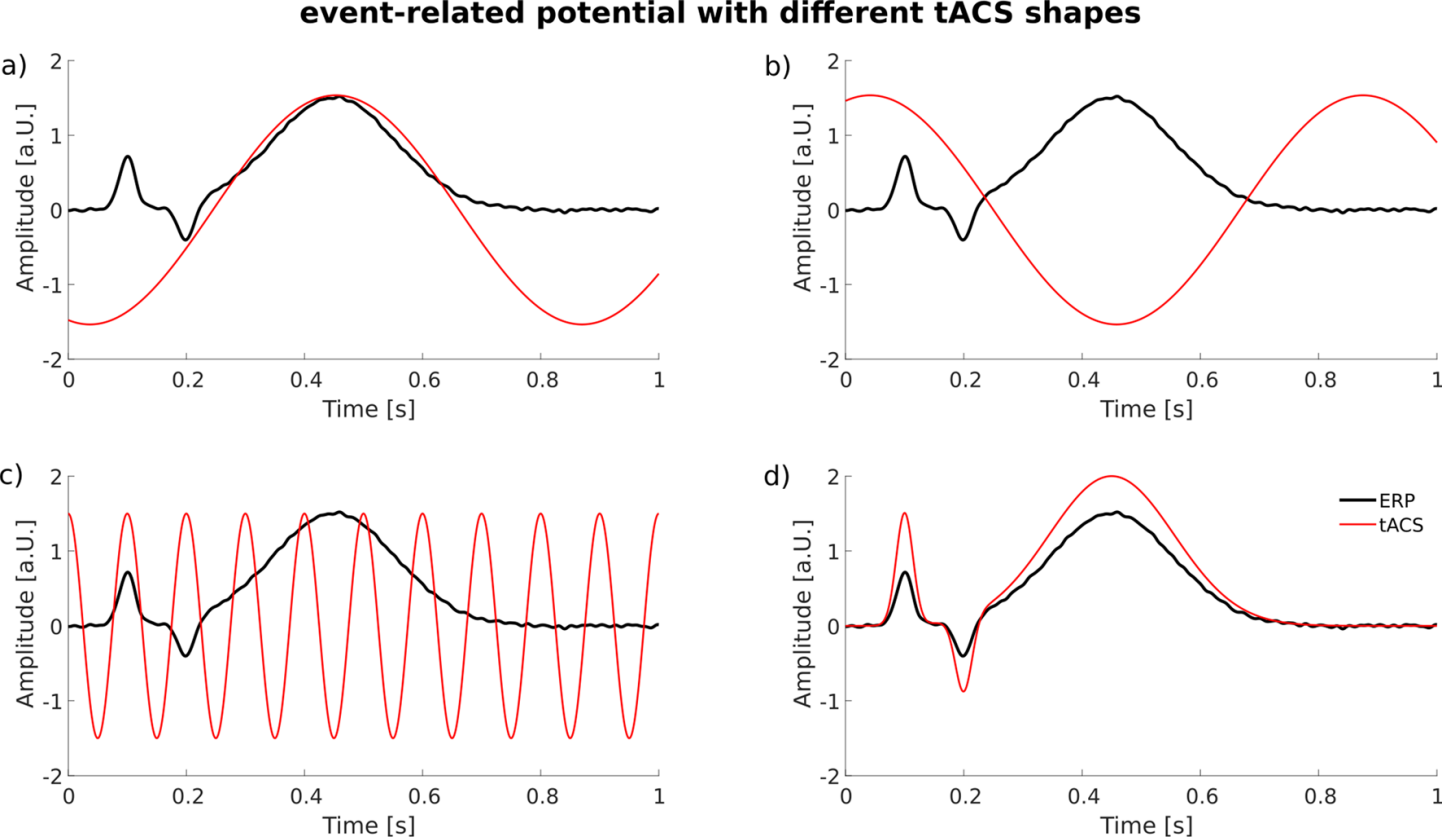
Future applications for ERP-aligned tACS. **a**) ERP-aligned tACS matching the peak of the P3 with the positive half-wave of the continuous tACS (as applied in the studies of (Boetzel et al. 2023; Dallmer-Zerbe et al. 2020; Kannen et al. 2022; Popp et al. 2019). **b**) ERP-aligned tACS matching the peak of the P3 with negative half-wave of the continuous tACS. c) ERP-aligned tACS matching the peak of the P1 with the positive half-wave of the continuous tACS in the alpha frequency range. d) Application of ERP-shaped tACS to modulate the entire ERP

In ERP-aligned tACS, the stimulation phase of the tACS is deliberately aligned with a stimulus presentation, so that a specific phase of the tACS (e.g., the peak) coincides with a specific component of the evoked ERP. This would have the potential to increase the power of the ongoing oscillations as well as the additive power that is evoked in each trial following the presentation of a stimulus. This method could be applied to all the components of ERP introduced earlier. For instance, it is feasible to apply continuous alpha-frequency-tACS while synchronizing the presentation of visual or auditory stimuli in such a manner that the peaks and troughs of the alpha stimulation align with the temporal characteristics of the P1 or the N1 components (see Figs. 1, 2, 3, 4, 5c). Notably, this method has previously been employed for selective modulation of the P3 component (Boetzel et al. 2023; Dallmer-Zerbe et al. 2020; Kannen et al. 2022; Popp et al. 2019). The following section will introduce four distinct studies in which this approach was implemented, followed by a comprehensive discussion of the diverse outcomes observed. The initial two studies, conducted by Popp et al. (2019) and Dallmer-Zerbe et al. (2020), employed individualized delta/theta tACS. In both studies, an identical paradigm was employed, with the only distinction being that Popp et al. (2019) investigated a cohort of healthy participants, while Dallmer-Zerbe et al. (2020) focused on a group of individuals diagnosed with attention deficit hyperactivity disorder (ADHD). Participants in both groups performed a simple visual oddball task (x-o-paradigm), and tACS was administered at an intensity of 1 mA, distributed across six electrode pairs. The stimulation parameters (latency and frequency of the P3 component) required for the individualized tACS application were determined using the data from the first experimental block without tACS. Remarkably, while both studies shared an identical experimental paradigm, only the ADHD patients exhibited significantly increased P3 amplitudes subsequent to stimulation as compared to the control group (Fig. 1b). Furthermore, a descriptive increase in pre-to-post delta/theta power was visible after stimulation in the patient group, contrasting with a decrease in delta/theta power observed in the sham condition. Moreover, individuals with ADHD demonstrated a reduction in omission-type errors (Dallmer-Zerbe et al. 2020). In contrast, Popp et al. (2019) could neither demonstrate a significant increase in the P3 amplitude within the stimulation condition as compared to the sham condition in healthy participants, nor a considerable power increase following tACS when compared with the sham condition. This raises the question of how these contradictory results could have occurred, despite the same paradigm being employed. Hence, we conducted an exhaustive investigation of both studies coming up with an improved paradigm (Boetzel et al. 2024) that could be used for a follow-up study aiming to enhance the P3 amplitude through ERP-aligned tACS (Boetzel et al. 2023). This improved paradigm was examined in two separate cohorts, including ADHD patients (Kannen et al. 2022) and a control group of healthy participants (Boetzel et al. 2023). One important modification compared to the studies conducted by Popp et al. (2019) and Dallmer-Zerbe et al. (2020) was made regarding the visual task. While Popp et al. (2019) and Dallmer-Zerbe et al. (2020) used a simple x-o-oddball task which led to a ceiling effect of performance in healthy humans, we developed a new paradigm which allows the performance to be reduced to an intended level, in that case to around 75% (Boetzel et al. 2024). The advantage of this stimulus material is that the difficulty level can be varied, while almost identical visual stimuli are used. Only the degree of the rotation of the stimuli (away from vertical) is changed between conditions. With this modulation of the rotation, artificially attenuated P3(m) amplitudes can be elicited in the hard condition. These results are depicted in Fig. 2, which shows the event-related fields (ERFs) of combined trials and targets and standards separately for two condition, the easy (rotation degree of 2°) and the hard (rotation degree of 0.5°) condition. The ERFs show a significant decrease in the P3(m) in the hard condition as compared to the easy condition (see Fig. 2). The behavioral data emphasize the effect of the task difficulty modulation, as the d-prime decreased and the reaction times for the standard trials increased significantly in the hard condition as compared to the easy condition. Thus, this visual task seems to be well suited to investigate the modulatory effect of ERP-aligned tACS, as 1) there was no ceiling effect in the performance and 2) the P3(m) amplitude was significantly decreased by the difficulty manipulation. These two factors were essential conditions for investigating the effect of ERP-aligned tACS, as when performance shows a ceiling effect, it cannot be further improved by the stimulation, and similarly, the neuronal response to the stimulus might already be at its maximum if the difference between stimuli (target and standard) is salient. Another parameter of the studies of Popp et al. and Dallmer-Zerbe et al. that was under investigation is the stimulation site for the ERP-aligned-tACS. Therefore, we conducted source localization analyses to identify the neural generators of the task-difficulty dependent P3 modulation, locating these generators in centro-parietal regions (Boetzel et al. 2024). Furthermore, the applied current intensity was increased, as the recommended threshold for electric field strength should be ~ 0.2 V/m in the target region (Krause et al. 2019; Reato et al. 2010), which was not reached in the studies by Popp et al. (2019) and Dallmer-Zerbe et al. (2020) as they divided the 1 mA current intensity to six electrode pairs. The reported findings together with the increased current intensity have been assimilated into subsequent research (Boetzel et al. 2023; Kannen et al. 2022), employing a slightly altered electrode montage and a the task that was successfully established. Interestingly, Boetzel et al. (2023) and Kannen et al. (2022) also presented findings that appear to be in contradiction. While Kannen et al. (2022) could not demonstrate an increase in P3 amplitude (see Fig. 3) or a power enhancement following the application of individualized delta-frequency, Boetzel et al. (2023) reported the presence of both effects—a P3 amplitude increase and an elevation in evoked delta power—subsequent to the administration of individualized delta tACS (see Fig. 4). It has to be noted, that Kannen et al. (2022) observed a significant stimulation effect concerning a late negative deflection denoted as the N700, an outcome that was not expected. Interestingly, both studies failed to demonstrate any stimulation effect on behavioral outcomes.

The interpretation of the outcomes of these four studies presents a challenge, given that even the improvements in study design failed to yield consistent results. Nevertheless, it is noteworthy that both recent studies (Boetzel et al. 2023; Kannen et al. 2022) found a stimulation effect resulting from delta tACS. Notably, in the case of Kannen et al. (2022), the observed effect occurred later in time course than initially expected. Drawing a comprehensive and well-founded comparison among these four studies is a complex endeavor, primarily due to variations in the visual task employed, differences in stimulation intensity, and variations in the study populations.

## Future Applications

These contradictive findings emphasize, once more, the critical significance of selectively adjusting single parameters in follow-up studies to exert precise control over the causal relationship between distinct parameters and observed results. One conceivable approach would involve the implementation of a study involving both ADHD patients and a control group, employing an identical paradigm yet including refined parameters. This would require the utilization of identical hardware, task design, and stimulation intensity across both cohorts and thereby facilitating a direct comparison. While these studies are of limited help in further understanding the relationship between EROs and ERPs, an initial step in examining the relationship between oscillatory activity power and, for instance, the P3 component, could involve the establishment of a paradigm that combines in-phase and anti-phase ERP-aligned delta tACS and sham tACS. With this approach, it is anticipated that the total power of delta oscillatory activity would exhibit an increase. Subsequently, it could be investigated whether the precise synchronization of the P3 peak with the peak (or trough; see Fig. 5a & b) of the sinusoidal stimulation waveform exerts any influence on the P3 amplitude. This is because it has not yet been demonstrated whether ERP-aligned tACS exhibits increased stimulatory efficacies compared to normal tACS, as it was shown that even tACS without a synchronization between the ERP component and the tACS wave is able to modulate the component respectively (Hu et al. 2021; Nakazono et al. 2020; Pahor and Jaušovec 2017). Another conceivable strategy under consideration includes the development of a stimulation protocol that incorporates the spatiotemporal characteristics of the entire ERP (see Fig. 5d). Consequently, this protocol aims to elicit modulatory effects on the ERP by applying event-related tACS rather than continuous tACS. Therefore, it is necessary to consider the frequency, latency, and spatial distribution of individual ERP components, thereby enabling the tailored application of ERP-shaped tACS. On the behavioral front, the evaluation of performance in a near-threshold visual or auditory detection task may be employed to assess the potential enhancement of participants’ stimulus detection abilities by ERP-shaped tACS.

## Conclusion

The targeted modulation of the ERP via transcranial alternating current stimulation is overall sparsely researched with only nine studies reporting on effects of classical continuous-tACS and only four studies employing the ERP-aligned method. In general, the published results are encouraging, but of course we cannot not rule out a publication bias towards positive outcomes. Overall, the effectiveness of modulating ERP-components seems to be highly task dependent. It does not seem like any component could simply be altered by administering a fitting stimulation frequency. The superiority of aligned tACS over non-aligned tACS, although theoretically plausible, has so far only limited evidence, due to the current absence of a direct comparison of both stimulation forms on an identical task.

## Data Availability

No datasets were generated or analysed during the current study.
